# Digitalization in orthopaedics: a narrative review

**DOI:** 10.3389/fsurg.2023.1325423

**Published:** 2024-01-11

**Authors:** Yasmin Youssef, Deana De Wet, David A. Back, Julian Scherer

**Affiliations:** ^1^ Department of Orthopaedics, Trauma and Plastic Surgery, University Hospital of Leipzig, Leipzig, Germany; ^2^ Orthopaedic Research Unit, University of Cape Town, Cape Town, South Africa; ^3^ Center for Musculoskeletal Surgery, Charité University Medicine Berlin, Berlin, Germany; ^4^ Department of Traumatology, University Hospital of Zurich, Zurich, Switzerland

**Keywords:** orthopaedics, telemedicine, digitalization, AI, machine-learning, robotics, sensor technology, smart implants

## Abstract

Advances in technology and digital tools like the Internet of Things (IoT), artificial intelligence (AI), and sensors are shaping the field of orthopaedic surgery on all levels, from patient care to research and facilitation of logistic processes. Especially the COVID-19 pandemic, with the associated contact restrictions was an accelerator for the development and introduction of telemedical applications and digital alternatives to classical in-person patient care. Digital applications already used in orthopaedic surgery include telemedical support, online video consultations, monitoring of patients using wearables, smart devices, surgical navigation, robotic-assisted surgery, and applications of artificial intelligence in forms of medical image processing, three-dimensional (3D)-modelling, and simulations. In addition to that immersive technologies like virtual, augmented, and mixed reality are increasingly used in training but also rehabilitative and surgical settings. Digital advances can therefore increase the accessibility, efficiency and capabilities of orthopaedic services and facilitate more data-driven, personalized patient care, strengthening the self-responsibility of patients and supporting interdisciplinary healthcare providers to offer for the optimal care for their patients.

## Introduction

Digital tools and applications were developed and implemented at a fast pace in the field of orthopaedic surgery in the past years and are beginning to shape this medical field on all levels—clinical and logistic processes, patient care, research, and education. Examples of technologies and digital tools that thrived in the past years are the Internet of Things (IoT), next-generation telecommunication networks, artificial intelligence (AI), big data analytics, blockchain technologies and sensors. These technologies have greatly changed the possibilities in healthcare provision by supporting and amplifying human cognitive functions and decision making (1). They are highly connected and inter-related and in combination, can contribute to the formation of digital ecosystems. Digital applications that are already implemented in healthcare systems among others are electronic health records, telemedical solutions, robotic assisted surgeries, three-dimensional (3D) modeling, virtual simulation, and visualization. Their use can improve the quality (accuracy and efficiency), accessibility and capability in the provision of services in the field of orthopaedic surgery (2). Furthermore, digital tools can enable the provision of more personalized and patient centered healthcare (3). The incorporation of AI systems in orthopaedic surgery can be attributed to emerging advancements in technologies based on navigated, computer guided and robotic assisted input (4). Interactive, virtual 3D-imaging (in conjunction with robotics) are replacing standard two-dimensional (2D) imaging modalities which has improved pre-operative planning and intra-operative functionality and therefore patient outcome. This type of “digital medicine” is especially evident in spine surgery with e.g., image-based pedicle screw placement where a robotic guidance system including pre-operative planning software is used. This can facilitate the placement of pedicle screws, specifically in patients with significant spinal deformities or alterations in anatomical landmarks (congenital malformation, degeneration, tumours, trauma, revision surgery) and thereby mitigating risks and complications (5).

The Covid-19 pandemic has contributed significantly to the accelerated use and implementation of digital tools and applications for direct patient care in the form of telemedicine (6). Due to the strict contact restrictions, it was a necessity to switch from conventional face-to-face appointments to telemedical applications and services. AI applications were used in the form of contact-tracking and rehabilitation apps and for the prediction of disease and resource burden of individual hospitals and regions. As digitalization will play an increasingly important role on all levels of health care provision and therefore also in the field of orthopaedics, having a fundamental understanding of current developments in this field is of great importance.

The following overview presents current developments of digital applications in the field of orthopaedic surgery and will examine their future applications and potential limitations and hazards.

## Methods

**Figure 1:**
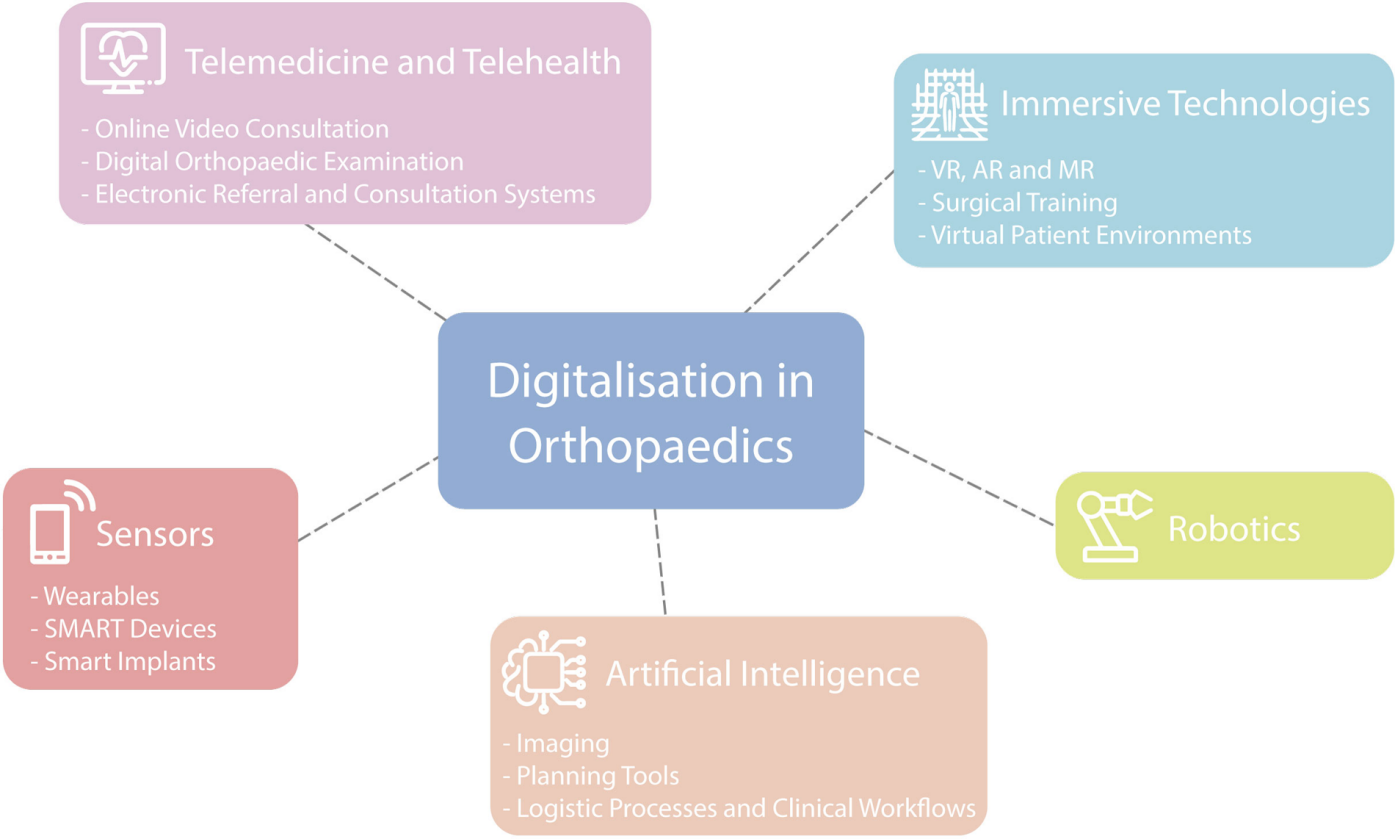
Overview of possible digital applications in orthopaedic surgery.

Key areas of digitalization in the field of orthopaedic and trauma surgery have been defined and summarized into separate subject areas by the authors, based on clinical experience and after evaluating published research of leading figures in the field (7). Subsequently, current literature on the topic was reviewed and discussed by the authors. The topics have not been presented in the manuscript with any rating of importance. Figure 1 is representing the various digital health applications used in orthopaedics which are discussed in this overview.

### Telemedicine and telehealth

Telemedicine is the distribution of medical (diagnostic, treatment, and follow-up care) services using telecommunication technologies. Telehealth on the other hand also includes other serviced outside of the physician-patient relationship (3). The central part of telemedicine are online video consultations including digital clinical examinations and electronic consultation and referral systems.

#### Online video consultations (OVCs)

Orthopaedic patients can be assessed remotely, especially for chronic conditions, using OVCs (8). OVCs show comparable results regarding patient satisfaction and patient related outcomes (PROMs) and have economic advantages (e.g., travel cost reduction)*.* Video consultations have been shown to be especially effective in the outpatient setting for the initial assessment of non-acute conditions including patient history and digital assessment of Range of Motion (RoM) as well as functional assessment. OVCs can also be used in post-surgical follow-up examinations including wound and RoM assessment, rehabilitation visits and private patient-doctor-discussions (9–11). It has been shown that OVCs are especially useful in times where inter-personal contact should be limited, as during the Covid-19 pandemic. Furthermore, OVCs are a good alternative for patients in none-acute settings who are from rural parts with limited hospital access (12).

Telehealth (including OVCs) and mobile health (mHealth) technologies such as sensors, wearables, and mobile applications, with the incorporation of AI and ML algorithms for data analysis, have enabled remote patient monitoring post-operatively with more efficient use of healthcare resources, decrease in health-related costs, and improved patient outcomes compared to current traditional post-operative interventions (13). The application of these technologies in the orthopaedic post-operative setting can provide the orthopaedic surgeon with remote, objective, and quantitative data, which can help them to make more (timeously) informed treatment decisions as well as continuously monitor patient progress and rehabilitation outcomes (14).

However, a few limitations of remote telemedicine must be noted. Firstly, patients may not have access to devices with compatible software for video consultations, or they may not be comfortable using the required technology, especially in the elderly population. In addition, both patients and health care providers need a stable and reliable internet connection. This might be a problem especially in neglected and rural areas (15). Secondly, digital patient encounters are more susceptible towards privacy and security risks. Ensuring secure platforms and practices is crucial to protect patient confidentiality and comply with healthcare data protection regulations. In addition to that, there are regulatory barriers to telemedicine as well as lacking regulations for necessary hard- and software, patient and data security, health insurance and reimbursement (16). Furthermore, as physicians have the potential possibility to provide medical services across geographic borders, multistate licensures must be established (16).

#### Digital orthopaedic examination

An important aspect of the OVC is the clinical examination of patients, which is composed of inspection, palpation, and a functional examination. The clinical examination and synopsis of the patient`s history is crucial for the initiation of further diagnostic steps.

Inspection is limited to visual impressions and can therefore be supported by digital media for data transmission. Palpation in a digital setting is currently still limited to self-palpation by the patient. Hereby, instructions by the examiner are of great importance. In addition to that, constant communication between physician and patient is important to inquire for changes in sensation, pain, or consistency in the examined region.

The digital transmission of tactile impressions has been subject of technical developments for many years (17). Remote palpation has only recently emerged as an independent research topic as the technological prerequisites for the direct cutaneous mediation of haptic impressions, in contrast to the mediation of haptic and tactile impressions in the sense of force feedback via surgical instruments, only became possible with the concept of “wearables” as a mediating technology between the human senses and the environment (18).

While passive mobility tests require professional support and guidance, the active ROM can be inspected by visual digital media. Consequently, the digital devices can be used to objectify clinical findings as well as rehabilitation performance and subsequently also to compare the individual patient with a historical collective (19–21). Technologies for real-time, marker-free motion capture can currently only work in simple motion patterns, as polyaxial movements can only be identified with limited accuracy (22).

In addition to that, several studies have described fully digital alternatives for the functional examination of the musculoskeletal system (23). Most of the examinations are adaptations of classical functional tests that can be performed by the patient without any external human support. To support this guided self-examination everyday objects, like tin cans and towels can be used to apply force and guide directions (23). Those objects can help to apply force or guide motion into a specific direction during the examination. Illustrations can be supportive in the preparation of the examination as they can visually explain the examinations and support the verbal instructions of the physician (24). The validity and reliability of the described digital examination alternatives is however not yet fully assessed and should therefore be prioritized as a research subject in the future (23). A limited number of studies has aimed to directly compare face-to-face functional examinations with digital alternatives in the online video consultation. It was shown that there is high accordance for inspection and ROM-testing, but palpation and the functional examination are less consistent in the digital examination (23).

#### Electronic referral and consultation systems

Access to specialist care is vital for a coordinated and efficient diagnosis and the treatment of patients. Traditionally, referrals and specialist consultations are performed by telephone or on paper. All those methods can lead to adverse events and medical errors during patient care, due to incomplete, fragmented, or unstructured communication and information exchange between different healthcare providers.

Electronic referral and consultation systems have been introduced to reduce waiting times and improve the access to specialist care (25). In addition to that, digitalizing these processes can improve their quality and completeness. Electronic referrals and consultations can enhance the communication and seamless information/data exchange between clinicians and therefore increase patient safety. Finally, electronic referrals and consultations can improve patients` experience and satisfaction of the referral process (25, 26).

A successful example for the implementation of an electronic referral system is the app Vula Mobile (Mafami Pty Ltd), which has been used since 2014 to refer patients to emergency centers and outpatient departments It has improved the quality and coordination of patient care within the healthcare system (27). Another example from is the introduction of an electronic referral system within a musculoskeletal model of care in Ontario (26). In comparison to the paper-based referral forms, electronic referral forms were more legible and complete and had significantly shorter processing times (26). In addition to that, this system is resource efficient and administrative requirements are minimized.

### Sensors

Sensors are devices that can detect changes in the physical environment (e.g., pressure, temperature, motion) which are converted into an electronic output which can be read by humans. Sensors are therefore a gap between the physical and digital world.

The use of sensors allows for objective, continuous and long-term monitoring of different patient specific parameters. A cross sectional survey study found that most participants would use a home-based automated digital measurement system with respect to post-operative follow-up; and another found that most would accept the use of a mobile application where personal health-related data is collected for post-operative monitoring (28, 29). Therefore it seems that overall, data protection is not a big concern.

#### Wearables

Traditionally, injury prevention in orthopaedics has been embodied with the aid of biomechanical assessments using kinematic and kinetic quantitative parameters with the aim to identify individuals at risk of specific injuries and to deliver feedback on prevention of high-risk movement patterns (30, 31). An increasing number of patients use wearables in their daily life and sensors can be used in different areas of patient care. Wearables used in the field of orthopaedic surgery are smartwatches, fitness trackers and motion as well as pressure sensors (32). Other examples for applications used are portable sensors such as inertial measurement units (IMUs), depth cameras, red-green-blue (RGB) cameras and electromyography (EMG) (30). This generation of big data with the integration of machine learning (ML) allows injury risk stratification. Further future applications include fall prevention systems. These systems include the use of wearable feet pressure sensors integrated with ML models to detect arrhythmic variation in phase distribution and unequal load distribution in gait analyses, which could alert via an application and therefore preventing a forthcoming fall and subsequent injury (33).

#### SMART devices

The interplay of better sensor technology and technologies like AI, big data analysis and ML have also allowed the development of Self- Monitoring Analysis and Reporting Technology (SMART) orthopaedic devices (34, 35). These devices, like braces, prosthetics and implants with embedded sensors can measure movement, force, and posture, aiming to improve and individualize patient care (36). For the upper extremity, wearable sensor settings have shown to give relevant data on patients rehabilitative outcome after surgery, e.g., in humeral head fractures (37). The use of such telehealth technologies is advantageous in postoperative monitoring, not only with the ability to reach more patients and reduce costs but is also particularly applicable to patient populations in remote areas (38).

#### SMART implants

One specific use of SMART devices in orthopaedics are smart implants (SI), which can be used in the assessment of fracture healing and for the detection of aseptic loosening in total joint arthroplasty, periprosthetic infections and other infections of the musculoskeletal system (39–41). Assessing the stage of fracture healing is crucial to provide patients with an adequate post-operative plan in regards of RoM allowance and weight-bearing restrictions and to detect non-unions early (42). The AO Fracture Monitor has been introduced and studied preclinically on ten animals attached to a locking compression plate (LCP) bridging a tibial defect (43). The implantable data logger (attached to the plate) collects various fracture healing parameters which are transferred wirelessly to the patient`s smartphone which allows remote assessment of the treating physician. In addition to fracture care, SI have been shown to detect implant loosening and osteo-integration in total hip arthroplasty (THA) in experimental settings detecting mechano-acoustic waves and transmitting these to an external coil (44). In total knee arthroplasty (TKA), strain gage-based load cells of the tibial component can be used to understand intra-operative biomechanics to determine alignment and implant sizes as well as to plan the post-operative care and rehabilitation scheme (36, 45). Several studies have been investigating spinal fusion procedures using strain sensors over the fusion rods to monitor progression of spinal fusion. However, these systems are not commercially available yet (46, 47). Another application are microelectromechanical systems (MEMS) based sensors attached to the implant, which can detect the presence of bacteria, before biofilm formation, detecting specific bacterial compounds (48). Other sensor technologies can detect active infections by detecting pH-changes, oxygen-levels and temperature and therefore also allow for monitoring of antibiotic treatment (49).

### Robotics

Robotics in orthopaedic surgery can be divided into two categories: haptic and active systems. Haptic navigation systems are passive, synergistic, surgeon-guided, and augment manual movement along a planned trajectory using “virtual fixtures” to improve outcomes. For example, quantitative TKA surgery is performed with advanced soft tissue balancing in real-time with the use of a navigation system to visualize, plan and control all the cutting steps and their effects on soft tissue (50).

Active robotic systems are fully automated, based on a preoperative plan and are carried out without any intervention of the surgeon (51, 52). An application is the planning of the femoral component in cementless THA. These procedures are however still associated with prolonged operating times (technical complexity, set-up time, etc.) (52).

In the future, “telemanipulated” master-control, slave-robot systems, could play an important role in orthopaedic surgery. These disengage the surgeon physically from the patient using a console providing information (the 3D surgical field) to the surgeon who then uses master controllers that filter, scale and translate the movements of the surgeon's hands to robotic arms (output) with significant assistance in tremor reduction in minimally invasive surgery (53). This type of system could be applied, specifically in remote, hard to reach sites like minimally invasive arthroscopic procedures. However, one of the major obstacles of this type of system is that there is no haptic feedback channel to provide force or position information or potentially augmented information such as planned trajectories (51). This type of robotic system could drastically revolutionize orthopaedic surgery in terms of minimal surgical access, avoidance of critical anatomical structures, improved accuracy of alignment, reduction in workload for the surgeon regarding ergonomics as well as less exposure to radiation and ultimately improved patient outcomes. However, it must be noted that integrating robotics into clinical and surgical workflows may necessitate additional time and resources, potentially leading to a temporary decrease in surgical efficiency during the initial learning curve as surgeons and healthcare staff require specialized training to operate orthopedic robots effectively (54, 55).

### Artificial intelligence

Artificial intelligence (AI) refers to the performance of tasks, that normally require human intelligence such as visual perception, speech recognition and decision-making, by computer systems (56). In the past years applications using artificial intelligence have greatly shaped the healthcare system.

#### Imaging

Imaging in orthopaedics is crucial for the detection and classification of fractures and the diagnosis of musculoskeletal disorders. Imaging is therefore important for the determination of treatment plans, intra-operative controlling, and the monitoring of post-operative outcomes, as well as for the detection of potential complications. The evaluation and interpretation of images is however highly subjective and dependent on many factors including the individuals experience and competence.

The use of 2D plain radiographs and 3D-imaging modalities such as Computerized Tomography (CT), Magnetic Resonance Imaging (MRI), as well as nuclear and molecular imaging, have become routine examinations in orthopaedic surgery. Recent advances in digital technology have seen a growing application of these imaging modalities in conjunction with AI, ML and deep learning (DL) (57–59).

The use of these integrated technologies can enhance the efficiency and accuracy by which pathologies of the musculoskeletal system are detected. Various studies have shown that DL in plain 2D radiographs has comparable accuracy in detecting and classifying fractures when compared to clinicians (60). Similarly, several ML and AI technologies have been shown to be superior in detecting and staging osteoarthritis of the hip and knee compared to trained radiologists (61). In addition to that, ML-trained AI programs are better in detecting implant loosening on 2D radiographs than experienced orthopaedic surgeons and can identify the type of implant satisfactory (61). The automated detection of spinal pathologies using ML-systems also shows good specificity and sensitivity (61).

In addition to that, CT- or MR-imaging can be used to three dimensionally reconstruct a patient`s individual anatomy, which allows to produce patient specific implants and the development pre-defined cutting guides. This also poses an important step towards more personalized treatment in the field of orthopaedic surgery. Those individualized implants are created using digital printing technologies.

Patient specific implants technology aims to reduce surgical time and to improve patient outcomes. Patient specific implant technology is already used in THA and TKA and corrective osteotomies. It has however also gained popularity in shoulder arthroplasty and ankle joint surgery (62). But it must be noted that long-term studies assessing the clinical efficacy of personalized orthopaedic implants are currently lacking and are needed before the widespread application patient specific implant technology is fully supported.

However, it is important to note that AI algorithms are influenced by the quality of the provided training data and are susceptible to biases present in the respective data. Therefore, prediction made by AI, especially for small cohorts, might be suboptimal because of their underrepresentation in clinical datasets. This can perpetuate and exacerbate disparities in healthcare outcomes (63). Furthermore, there are also concerns regarding data and privacy security of patients in the use of AI-technology (64). Finally the “black box” nature of some AI models hinders their interpretability, making it challenging for healthcare professionals to understand the rationale behind specific AI-generated recommendations (63).

#### Planning tools

Three-dimensional imaging (i.e., CT) and digital pre-operative planning tools can enhance the execution of surgical approaches, including reduction and fixation in the treatment of fractures. For example, digitally planned surgeries of pelvis fractures, show superior outcomes when compared to conventional 2D methods (65). Digital planning tools are also available for long bone fixation, arthroplasty procedures, spine deformity correction surgery and post-traumatic deformity correction procedures (66). AI-based planning software in knee and hip arthroplasty has been shown to be superior compared to the manufacturer`s software with less intra-operative corrections made by the surgeon (67). Ultimately, several studies have shown great accuracy in predicting outcomes of bony pathologies based on radiological imaging using AI and ML methods for general fracture treatment, arthroplasty, and spine deformity correction, and can be used to aid in surgical planning to reduce short-term as well as long-term complications (61, 68).

#### Logistic processes and clinical workflows

In life-threatening (orthopaedic) emergencies, the correct clinical assessment, choice of the target hospital and transport method for the patient are crucial (69). All of those preclinical aspects can be optimized by digital tools and applications, which aim for faster resource allocation and early appropriate assessment of the clinical situation. ​Assessment and outcome scores can be used as decision-making aids for initiating out-of-hospital treatment, situation-dependent choice of adequate hospital and the automated and improved communication with trauma centers (70). Different scoring systems have been implemented to facilitate preclinical decision support (71, 72). The flow of information can be optimized through real-time telemetry and automated data processing between the emergency services and the hospital; and furthermore, hospital capacities can be translated to control centers via automated systems to ensure optimal patient triage and distribution (70, 73).

ML based systems for non-invasive prediction of impending complications and indications for on-site treatment or immediate action in the target trauma center have been shown to achieve similar or better results than “raw” experience (71, 72, 74).

Approximately 8% of all deaths due to major (orthopaedic) trauma are considered potentially preventable (75). The connection of computer-generated stimulations through visual and auditory displays during the resuscitation can enhance trauma care professionals' interaction and might reduce faulty omission and miscommunication (75). Previous studies have shown effective tools for the prediction of injury pattern. Probabilistic graphic models in conjunction with CT-3D-reconstructions and trauma victim`s vital parameters to predict outcomes based on the location of penetrating injuries have been shown to be an effective tool to increase time efficiency and safety in the treatment of patients with penetrating injuries (76). Several AI algorithms can be used to detect arterial injuries using specific patients` parameters (77). Audio analysis of spoken words in the resuscitation room can be used for data collection and categorization of the resuscitation phases (e.g., arrival of patient, primary survey, secondary survey) (78). Machine-learning tools and artificial neural networks (ANN) have been used to develop several systems like smartphone applications and ensemble classifiers as decision tools for the prediction of hemorrhage or need for blood transfusions, including mass transfusion protocols (72, 79, 80). Furthermore, AI has been shown to have a better classification accuracy for the hemorrhage intensive severity and survivability score (HISS) in the clinical setting and can be used as an adjunct to the score itself (81). Amongst other ML algorithms and networks, the AI-based TraumaAID computer program has been shown to be helpful in the prediction of need for emergency interventions (82–84).

Several ML and ANN systems have been shown to perform with higher accuracies than established outcome scores (85, 86). The WATSON Trauma Pathway Explorer (IBM), a machine-learning prediction tool, has been validated and shown to outperform the TRISS regarding early mortality. Furthermore, the application can predict sepsis as well as SIRS more accurately than other existing physiological scoring systems (87). Big data systems, ML and ANN can facilitate decision-making in the acute setting in poly-traumatized patients and further have the potential to improve or replace established scoring-systems and may build the basis for personalized medicine in severely injured patients.

Digital tools have also evolved in the orthopaedic outpatient sector, especially during Covid-19. Telemedical platforms for the triage of patients to specialty providers and to distinguish chronic conditions from urgencies and emergencies have been implemented to optimize resource allocation (7). Chatbots using AI technology were shown to be very useful for the triage of patients to the correct providers and can ease personnel shortages (7).

In addition to that, orthopaedic surgery is profiting from digitally enhanced operating rooms known as hybrid operating rooms. Hybrid operating rooms are aseptic environments that combine the traditional operating room with advanced imaging systems (CT or MRI). This allows for real-time 3D imaging of the patient during the surgical procedure without changing the location. This means that diagnostic and therapeutic procedures can be carried out simultaneously. Generally, hybrid operating rooms are run by multidisciplinary teams consisting of surgeons and radiologists. This offers the possibility to perform complex, image-guided, conventional, and minimally invasive procedures. In orthopaedic surgery, applications include spine and pelvis surgery (88). Using intraoperative 3D imaging increases the accuracy of operative procedures (e.g., screw placement) and operation errors can be detected an early stage. This, in turn, can reduce the rate of revision surgeries (89). However, it must be noted that the radiation exposure for staff within the hybrid operating room can be high and it is important to avoid excessive radiation exposure by adhering to radiation protection measures (90).

### Immersive technologies—virtual, augmented, and mixed reality

The developments and advancements of immersive technologies have the potential to change the provision of healthcare within orthopaedics (91). It can be differentiated between three types of immersive technologies: virtual-, augmented- and mixed reality. In virtual reality users immerse into a simulated three-dimensional computer-generated environment. This means that a fully digital simulation of a real environment is created while the real world is fully hidden. Virtual reality applications involve a head-mounted display and two handheld devices for placing the user into the simulated environment and to convey visual and physical feedback. In general, three forms of virtual reality applications can be defined: non-interactive simulators, interactive simulators with visual feedback and interactive simulators with haptic feedback (92). In augmented reality, virtual objects are overlaid onto the real world. This means that rather than creating a fully synthetic digital environment the real-world is supplemented with digital sensory input. Mixed reality connects virtual objects into the real world. This means that the real world and the digital world are blended, and physical and digital objects coexist and interact in real time.

#### Immersive technologies and surgical training

The use of immersive technologies can be enriching tools in medical education, especially in the field of surgery. In the past years, there have been drastic changes in western healthcare systems and in the provision of healthcare which do not only include more sophisticated surgical techniques, increased focus on administrative and other non-clinical tasks and work hour restrictions, but also greater sensitivity and interest in patient safety and higher expectations of surgical outcomes (93, 94). All of these developments increase the demands on residents of surgical faculties and hinder the surgical training and practice of surgical techniques in the operating room. Immersive technologies might be used as eligible alternative for surgical training as it allows the unlimited and patient-safe practice of surgical processes and surgical techniques. The implementation of immersive technologies in residency training could present as feasible alternative for real-world surgical training. This approach is especially appealing in times of less frequent patient and operating room exposure by residents, for example during the Covid-19 pandemic (95).

Recent studies suggest that the use of virtual reality tools in residency training has the potential to improve and translate surgical skills into the operating room (96). Furthermore, virtual reality also allows for a standardized and objective evaluation of different parameters during training, including the accuracy of the surgical technique or the time taken for different surgical steps (97).

Especially in the practice of arthroscopy using virtual reality has been of great interest and has become a focus of research in the past years. Arthroscopic simulators can not only present 3D anatomy, mimic surgical tools and pathologies but can also simulate realistic events including cartilage damage or bleeding. In addition to that, the trainee's performance can be analyzed and suggestions for improvement can be proposed (98). Previous studies could show that virtual reality simulator training can advance the basic arthroscopic skills and decrease surgery times of residents (99). Walbron et al. investigated the effect of virtual reality arthroscopy training on arthroscopy skills among 107 first year residents and found that there was a significant improvement in camera alignment and path trajectory (100).

Other possibilities of virtual reality application in orthopaedic surgery residency training include the practice of intramedullary nail and pedicle screw placement, arthroplasty, and fixation of fractures. It was shown that immersive virtual reality is better in the mediation of technical and non-technical skills when compared to traditional learning methods in orthopaedic resident education (101).

However, there are barriers to the comprehensive and structured implementation of virtual reality into orthopaedic surgery residency programs. There is only limited experience and research on structured complementary virtual reality training during residency programs. Further, the high costs associated with VR and AR hardware, can diminish general access to these technologies.

Augmented reality tools like for example the HoloLens (Microsoft) can be used by medical students and professionals to view 3D anatomy models and to understand complex procedures and processes (91). Some studies have also described augmented reality applications in the field of arthroplasty as supplement to computer assisted surgery. Here, visual overlays can be projected on the real world by using pre-operative CT-scans or intra-operative landmarks. At present there is still limited evidence for the use of augmented reality in arthroplasty and clinical studies are lacking. In pre-clinical settings the use of augmented reality has however improved surgical accuracy and reproducibility and has contributed to less radiation exposure.

#### Virtual patient environments

Immersive technologies can also be used to create virtual patients' environments for patient rehabilitation and the provision of perioperative physiotherapy (7). Applying those technologies could facilitate more personalized and patient-centered rehabilitation. Physiotherapy and rehabilitation measures can be performed remotely with no need for travel and allow for continuous monitoring of the patient. In a systematic review and meta-analysis by Gumaa et al. traditional rehabilitation and virtual reality rehabilitation showed comparable results across several orthopaedic diagnoses in terms of functionality and pain (102).

## Discussion

During the last decade, there has been a rapid increase in the use of digital tools in the field of orthopaedic surgery. Past research has shown that the utilization of digital technologies could improve the accessibility, efficiency and capabilities of medical services and evoke timely and active interventions by physicians (103). Particularly orthopaedic digital care could provide more data-driven, personalized care and can aid physicians with auxiliary diagnostic functions based on medical principles and data analysis models, thus prompting more efficient and effective diagnosis and treatment decisions ranging from prevention to rehabilitation. Research on orthopaedic digital medicine and its clinical transformation is rapidly developing (104). This could form the basis for more individualized and personalized medicine with both, strengthening patientś self-responsibility and supporting interdisciplinary healthcare providers to offer optimal care for their patients. Challenges that effect the successful implementation and integration of digital technologies into clinical routines include the lack of evidence based digital health standards as well as potentially reduced privacy, reimbursement regulation, licensing, and data governance regulations (105, 106). In addition to that, it is important to note that human factors as well as the acceptance and trust in technology and digital transformation from both a physician and patient perspective will presumably play an important role in the implementation of digital applications in the healthcare systems. Therefore, it will also be of great importance for health communities to make underlying ethical, political, human, and legal challenges subject of further discussions.
